# Uric acid as a predictor of endothelial dysfunction in patients with metabolic syndrome

**DOI:** 10.20945/2359-3997000000298

**Published:** 2020-10-21

**Authors:** Charanpreet Singh, Sanjay Jain, Veena Dhawan, Naveen Kalra, Savita Kumari

**Affiliations:** 1 Post Graduate Institute of Medical Education and Research Department of Internal Medicine Chandigarh India Department of Internal Medicine, Post Graduate Institute of Medical Education and Research, Chandigarh, India; 2 Post Graduate Institute of Medical Education and Research Department of Experimental Medicine Chandigarh India Department of Experimental Medicine, Post Graduate Institute of Medical Education and Research, Chandigarh, India; 3 Post Graduate Institute of Medical Education and Research Department of Radiodiagnosis Chandigarh India Department of Radiodiagnosis, Post Graduate Institute of Medical Education and Research, Chandigarh, India

**Keywords:** Uric acid, metabolic syndrome, endothelial dysfunction

## Abstract

**Objective:**

We conducted a study to examine the association of endothelial dysfunction and oxidative stress with uric acid levels in patients of metabolic syndrome.

**Subjects and methods:**

One hundred and two patients of Metabolic Syndrome (International Diabetes Federation definition) were included in the study. Anthropometric measurements, serum uric acid levels, fasting blood sugar levels and lipid levels, as well as malondialdehyde and reactive nitrogen intermediates were measured after an 8-hour fasting period. Flow mediated vasodilation (FMD) of the brachial artery was measured and endothelial dysfunction was defined as an increase in diameter < 10% post compression.

**Results:**

A total of 102 patients were included in the study. Mean uric acid level was 5.49 ± 1.61 mg%. A total of 59 patients in the study had endothelial dysfunction, defined by an abnormal FMD. Patients with an abnormal FMD had higher levels of serum uric acid which was statistically significant (p value = 0.010). Serum RNI and MDA levels were negatively correlated with uric acid, but did not reach statistical significance. Patients with an abnormal FMD had a lower RNI level, but this did not reach statistical significance. Serum MDA levels were significantly higher in patients with an abnormal FMD (p value = 0.038).

**Conclusions:**

Uric acid was significantly associated with endothelial dysfunction in patients with metabolic syndrome in our study. It was inversely correlated with serum RNI and MDA levels, but this did not reach statistical significance.

## INTRODUCTION

Metabolic syndrome (MS) is a universal health problem. A number of interconnected metabolic, biochemical, physiological and clinical factors interact and lead to the development of metabolic syndrome. Hyperuricemia is seen almost universally in these patients, but is not a part of the multiple criteria defining metabolic syndrome ( 1 - 3 ). Although it is considered to be an element of the overall spectrum of metabolic syndrome, newer evidence indicates that increased serum uric acid levels possibly predate the diagnosis of metabolic syndrome and may play a part in its pathogenesis ( 4 ). Further, uric acid has been found to be associated with endothelial dysfunction, oxidative stress and arterial stiffness – all factors associated with cardiovascular events. This association is often confounded by the presence of other risk factors, such as hypertension, diabetes etc. Through this study, we hypothesize that uric acid is an integral component of metabolic syndrome and may be an independent predictor of endothelial dysfunction and oxidative stress in patients with metabolic syndrome.

## SUBJECTS AND METHODS

One hundred and two patients with metabolic syndrome were included in the study from the outpatient department (OPD) of a tertiary care hospital in northern India. Ethical committee clearance was obtained from the hospital ethics committee before starting the study. Written informed consent was obtained from each patient before enrollment. Metabolic syndrome was defined according to the International Diabetes Foundation (IDF) criteria ( 5 ). Individuals above the age of 18 years and fulfilling the IDF definition were included in the study. Patients with a history of smoking, chronic kidney disease, heart failure, stroke and coronary artery disease were excluded from the study.

Five milliliters of venous blood was withdrawn after a period of overnight fasting. All patients underwent a thorough physical examination, including measurement of height, weight, BMI and waist circumference. Blood pressure was measured using a standard sphygmomanometer in a sitting position after a period of rest. Hyperuricemia was defined as > 7 mg% and > 6 mg% in male and female patients respectively.

Flow Mediated Vasodilation (FMD) was carried out for all patients as a measure of endothelial dysfunction utilizing the technique described by Celermajer and cols. ( 6 ). All measurements were carried out at the same location in the longitudinal section, 5-10 cm superior to the antecubital fossa of the right upper arm. The skin was marked at the end of the first stage of rest once a satisfactory location was found to carry out the study. After the marking of the skin, the arm remained in the same position throughout the study. Then, the diameter of the brachial artery was determined thrice in the first stage and the mean of the three values was used for further analyses. A blood pressure cuff was place over the arm and inflated to 300 mmHg which resulted in a complete cessation of blood flow. The pressure was maintained for 5 minutes after which the pressure was released. A 2nd and 3rd scan was obtained after fifteen and ninety seconds respectively after releasing the pressure. Ninety seconds after ischemia, 3 measurements of the diameter of the brachial artery were taken at the diastolic period. The mean of these measurements was used in the consequent analyses. FMD response was expressed as the change in end-diastolic diameter of the brachial artery during reactive hyperemia compared with the baseline (rest) value and used as a measure of endothelium-dependent vasodilatation. A change of < 10% in the diameter of the artery was used to define an abnormal FMD and hence, endothelial dysfunction ( 7 ). FMD measurement is not usually carried out at our center, and was conducted by a single individual for all patients of the study.

Fasting blood glucose, lipid profile and uric acid were measured using a sequential multiple analyzer. Blood levels for Reactive Nitrogen Intermediates were determined as a measure of endothelial dysfunction. 2 mL heparinised venous blood of the patient was centrifuged and the separated plasma was mixed with Griess reagent ( 8 ) (1:1 v/v 0.1% N-1, napthyl ethylene diamine in water with 1% sulfanilide in 5% orthophosphoric acid). This mixture was centrifuged and the absorbance measured at 546 nm afterwards with Vikon spectrophotometer. Nitrite concentration was devised from the standard sodium nitric acid curve.

Lipid Peroxidation product Malondialdehyde (MDA) was quantified in the blood as a marker of oxidative stress. 1 milliliter of patient serum was mixed with 2 mL of Trichloroacetic acid (TCA) – Thiobarbituric acid (TBA) – Hydrochloric acid (HCL) solution and heated for fifteen minutes in a boiling water bath. After cooling, the mixture was centrifuged at 3000 rpm for ten minutes after which the precipitate was removed. The absorbency was determined at 535 nm against reagent blank, which contained the entire reagent minus the serum ( 9 ).

Data was tabulated using Microsoft Excel and analyzed using Statistical Package for Social Sciences (SPSS Inc., Chicago, IL, version 22.0 for Windows). Measures of central location (mean, median) and measures of dispersion (standard deviation and standard error) were calculated for quantitative variables. For skewed data, Mann-Whitney test was used to compare the means of two groups, and Kruskal Wallis test was used for more than two groups. Qualitative or categorical variables were described as frequencies and proportions. Coefficient of correlation was calculated using Pearson and Spearman coefficient for normal and non-normal data respectively.

## RESULTS

One hundred and two patients were included in the study. Table 1 shows the baseline characteristics of the patients in the study. Baseline characteristics of the patients were compared between the male and female population in the study and were significantly different only in relation to height (p value = 0.000) and BMI (p value = 0.005).

**Table 1:** Baseline characteristics

Characteristic	Overall-102 *	Male-46 #	Female-56 #	P value
Height (in meters)	1.60 (1.36-1.83)	1.67 ± 0.08	1.54 ± 0.05	0.000
Age (in years)	51.83 (28-82)	50.09 ± 10.49	53.27 ± 11.54	0.425
Weight (in kilograms)	73.22 (48-110)	75.32 ± 12.42	71.48 ± 14.49	0.129
BMI (in kilograms/meter squared)	28.43 (20.7-42.6)	26.72 ± 0.58	29.82 ± 0.74	0.005
Waist circumference (in centimeters)	99.07 (83-124)	99.50 ± 8.64	98.72 ± 1.25	0.602
Uric Acid (in mg%)	5.49 (2.08-13)	5.80 ± 1.79	5.24 ± 1.41	0.200
Triglycerides (in mg%)	204.96 (68-1000)	209.70 ± 15.45	201.07 ± 19.18	0.323
HDL (in mg%)	45.89 (13.80-83.55)	43.80 ± 13.88	47.61 ± 13.74	0.109
FBS (in mg%)	124.12 (74.6-344.7)	123.36 ± 6.30	124.74 ± 6.15	0.514
LDL (in mg%)	121.62 (21.3-251.71)	120.33 ± 45.66	122.68 ± 43.11	0.600
HbA1C (in %)	6.71 (4.60-11.00)	6.87 ± 1.68	6.57 ± 1.44	0.499
Cholesterol (in mg%)	196.23 (77.0-372.97)	192.81 ± 57.20	198.99 ± 49.01	0.314
Hypertension	90 (88.2%)	49/56	41/46	0.799
Diabetes mellitus	50 (49%)	28/56	22/46	0.827

* Values are mean (range);

# Values are mean ±SD/SE.

**Figure 1:**
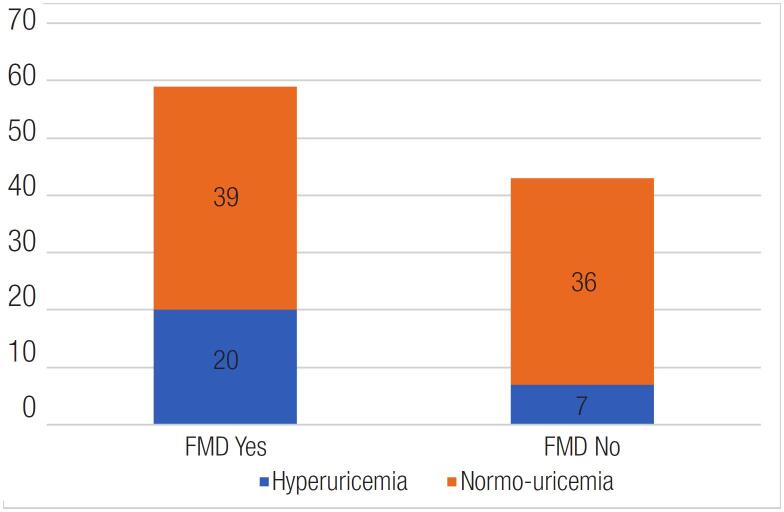
Distribution of abnormal FMD in patients according to serum uric acid levels

The mean serum uric acid was 5.49 ± 1.61 mg% in the study. Twenty-six point five percent of the study population had hyperuricemia, defined by the traditional gender-based cutoffs of 7 mg% and 6 mg% in males and females respectively. Hyperuricemia was associated with the presence of an abnormal FMD in a statistically significant manner (p value-0.046) ( Figure 1 ). Uric acid was inversely associated with the percentage increase in brachial artery diameter in a statistically significant manner (r = -0.265) (p value-0.002). It was also inversely correlated to serum RNI and MDA levels, but the correlation did not reach statistical significance. The other study clinical and biochemical parameters did not differ significantly between patients with and without hyperuricemia ( Table 2 ).

**Table 2:** Clinical and biochemical data of patients grouped according to Uric Acid levels

Characteristic	Uric acid normal (n = 27)	Uric acid raised (n = 75)	p value
Hypertension	25 (92.6%)	65 (86.6%)	0.509 *
Diabetes	14 (51.9%)	36 (48%)	0.731 *
Age (in years)	54.22 ± 13.51	50.97 ± 10.13	0.307 #
Height (in meters)	1.58 ± 0.10	1.61 ± 0.09	0.233 #
Weight (in kgs)	72.50 ± 13.75	73.47 ± 13.72	0.646 #
BMI (in kilograms/meter squared)	29.01 ± 5.59	28.21 ± 4.92	0.677 #
Waist circumference (in centimeters)	99.30 ± 9.40	98.99 ± 8.90	0.879 #
Flow mediated Vasodilation	7 (25.9%)	36 (48%)	0.046 #
Triglyceride (in mg%)	172.94 ± 11.0	216.49 ± 16.5	0.387 #
HDL (in mg%)	47.41 ± 2.79	45.35 ± 1.58	0.611 #
FBS (in mg%)	129.94 ± 11.15	122.02 ± 4.46	0.426 #
LDL (in mg%)	116.02 ± 7.75	123.65 ± 5.24	0.478 #
HbA1C (in %)	6.59 ± 1.45	6.76 ± 1.59	0.788 #
Cholesterol (in mg%)	187.31 ± 42.54	199.49 ± 55.77	0.266 #
RNI (in ηM)	84.08 ± 20.28	65.59 ± 7.04	0.585 #
MDA (in µM)	1.97 ± 0.12	2.17 ± 0.18	0.692 #

* Test applied is Chi Square Test;

# Test applied is Mann Whitney U test.

ηM: nano Molar; µM: micro Molar. Values expressed are median ± SD.

Fifty-seven point eight percent (n = 59) of the study population had an abnormal FMD, defined as a < 10% increase in brachial artery diameter after proximal occlusion. FMD was inversely correlated with uric acid levels (p value- 0.010) and serum MDA levels (p value-0.038) in a statistically significant manner. Serum RNI levels were lower in patients with an abnormal FMD, but this difference did not reach statistical significance. FMD did not correlate with the other co variables in a significant manner, including hypertension, diabetes, age, height, weight, waist circumference, lipid profile and number of components of metabolic syndrome. The relationship between FMD and the other variables in the study is shown in Table 3 .

**Table 3:** Clinical and biochemical data of patients grouped according to FMD

Characteristic	FMD Normal (n = 43)	FMD Abnormal (n = 59)	p value
Hypertension	36 (40%)	54 (60%)	0.227 *
Diabetes	23 (46%)	27 (54%)	0.441 *
Age (in years)	50.33 ± 11.12	52.93 ± 11.11	0.213 #
Height (in meters)	1.59 ± 0.09	1.61 ± 0.09	0.200 #
Weight (in kgs)	74.16 ± 14.50	72.52 ± 13.11	0.474 #
BMI (in kilograms/meter squared)	29.09 ± 5.09	27.93 ± 5.08	0.201 #
Waist circumference (in centimeters)	99.31 ± 9.69	98.90 ± 8.52	0.722 #
Uric Acid (in mg%)	5.06 ± 1.33	5.81 ± 1.74	0.010 #
Triglyceride (in mg%)	196.99 ± 23.53	210.77 ± 13.51	0.065 #
HDL (in mg%)	43.98 ± 13.83	47.28 ± 13.85	0.403 #
FBS (in mg%)	126.42 ± 5.54	122.44 ± 6.46	0.150 #
LDL (in mg%)	118.35 ± 6.86	124.01 ± 5.68	0.466 #
HbA1C (in %)	6.83 ± 1.57	6.67 ± 1.55	0.431 #
Cholesterol (in mg%)	191.58 ± 54.82	199.54 ± 51.24	0.486 #
RNI (in ηM)	77.36 ± 11.55	65.47 ± 9.76	0.403 #
MDA (in µM)	1.89 ± 0.09	2.28 ± 0.23	0.038 #

* Test applied is Chi Square Test;

# Test applied is Mann Whitney U test.

ηM: nano Molar; µM: micro Molar.

Subgroup analysis was performed on the basis of gender. In the female study participants, the number of components of metabolic syndrome (3 vs 4 vs 5 components) (p value = 0.01) and hypertension (p value = 0.003) were found to be associated with FMD in a statistically significant manner, in addition to hyperuricemia. This association was not seen in the male participants.

Mean RNI was 70.48 ± 7.44 µM and mean MDA was 2.12 ± 0.14 ηM in the study. Patients with endothelial dysfunction had a lower serum RNI level, although it failed to reach statistical significance (p value = 0.403). RNI and MDA levels were inversely correlated to each other in our study, however it wasn't statistically significant (p value = 0.488).

A logistic regression analysis was performed to ascertain the effects of uric acid, age, hypertension, diabetes, BMI category, waist circumference, triglyceride levels, HDL and MDA levels on the likelihood that patients had an abnormal FMD. A 1 mg/dL increase in uric acid was associated with a 1.44-time risk of having an abnormal FMD (p value 0.016) (95% CI 1.069 – 1.939).

## DISCUSSION

Higher uric acid levels were associated with a significantly lower increase in brachial artery diameter post proximal compression and were associated with an abnormal FMD in our study. This is in concordance to the results obtained by Huang and cols. in their study, in which FMD was negatively correlated with uric acid levels in individuals with MS ( 10 ). Zoccali and cols. also found a similar correlation in their study conducted in patients with hypertension ( 11 ).

The association between uric acid and endothelial dysfunction was seen only in male hypertensive patients with hyperuricemia in the study by Huang and cols. ( 10 ). This was contrary to our study, where the relationship between uric acid and endothelial dysfunction was present in the entire study population, and was more prominent in the female study population only. Various studies have tried to establish the variance in FMD among male and female populations. In the study by Skaug and cols., females below the age of 70 had a consistently higher increase in vessel diameter post compression as compared to age matched males ( 12 ). This is likely due to the difference in hormonal milieu between males and females. Estrogen plays a central role in endothelial dysfunction as evidenced by the fact that the sex difference in FMD diminishes once females reach the menopausal age group. Further, estrogen administration was shown to increase the FMD in postmenopausal females in the study by Lee and cols. ( 13 ). Studies have shown that uric acid correlates with cardiovascular mortality only in women and not in men ( 14 ). A possible hypothesis may link endothelial dysfunction due to hyperuricemia as a cause for this increased mortality.

We also found a higher number of components of MS to be correlated with the presence of endothelial dysfunction, although this relationship was present only in the female patients of the study population. Various studies have shown that an increase in number of components defining MS is linked with an increase in endothelial dysfunction ( 15 , 16 ). Endothelial dysfunction also correlates with individual components defining MS, including waist circumference and triglyceride levels ( 16 ). FMD did not correlate with waist circumference, diabetes, triglyceride and HDL as individual components in our study, although an overall increase in components of MS was associated with endothelial dysfunction.

Serum RNI levels did not correlate with uric acid in our study. In the study by Khosla and cols., hyperuricemic rats had lower levels of nitrates and nitrites in their blood ( 17 ). This difference may be due to a difference in the model adopted on which the study was conducted, i.e. humans vs mice. Increased oxidative stress correlates with the presence of MS and also with increased insulin resistance in these patients ( 18 ). Serum MDA levels also correlated with endothelial dysfunction in our study. However, serum uric acid levels didn't correlate with serum MDA. Krishna and cols. found higher serum uric acid and MDA levels in patients with pre-eclampsia, but uric acid and MDA levels had a weak correlation between themselves ( 19 ). This suggests that uric acid may be one of many factors causing increased oxidative stress seen in individuals with metabolic syndrome, though not corroborated by our findings. Oxidative stress has also been hypothesized as one of the mechanisms by which uric acid may be linked to the development of arterial stiffness.

Endothelial dysfunction and arterial stiffness both have been shown to be significant predictors of cardiovascular events ( 20 ). Maloberti and cols. studied the role of uric acid and presence of arterial stiffness in a hypertensive population in a longitudinal study ( 21 ). They showed that while uric acid was associated with a higher pulse wave velocity, it lost its statistical significance in regression analysis. A possible explanation was the presence of metabolic syndrome as a confounding factor, which was included in the regression analysis. Similarly, a recent meta-analysis has shown an association of serum uric acid levels and pulse wave velocity (both carotid-femoral and brachial-ankle) in the general population ( 22 ). Thus, while more robust evidence pointing towards clear causation is lacking, uric acid may play a significant role in the development of cardio-vascular complications through various mechanisms, such as endothelial dysfunction and arterial stiffness.

A major limitation of our study is the small study population. Another limitation of our study was the fact that factors such as duration of hypertension, and use of anti-hypertensive drugs which may influence endothelial function, as well as uric acid metabolism, were not taken into consideration.

In conclusion, a positive correlation was observed in the present study between endothelial dysfunction and serum uric acid and MDA levels. Uric acid is an independent marker of endothelial dysfunction in individuals with MS, and individuals with a serum uric acid value less than the traditional gender defined hyperuricemia cutoffs may also have endothelial dysfunction.

The likely mechanism for the endothelial dysfunction is due to a higher state of oxidative stress, as evidenced by the correlation between MDA levels and an abnormal FMD. Though uric acid levels didn't correlate with MDA, uric acid is known to cause oxidative stress. Further research is needed to investigate the relationship between uric acid and other markers of oxidative stress in patients with MS, including ADMA and reactive oxygen species. Research is also needed to see whether treatment of asymptomatic patients with elevated uric acid levels will impact the development of endothelial dysfunction in patients with MS, and hence improve cardiovascular morbidity and mortality associated with the syndrome.
